# Supplementary figures and images for: Regional variations in Helicobacter pylori infection, gastric atrophy and gastric cancer risk: The ENIGMA study in Chile
Source: PLoS One. 2020 Sep 8;15(9):e0237515. doi: 10.1371/journal.pone.0237515 (PMC7478833; doi:10.1371/journal.pone.0237515)

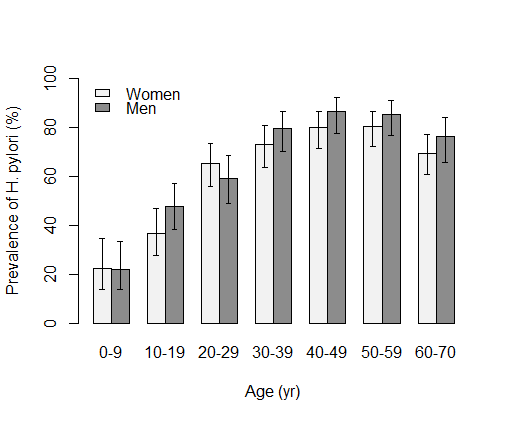

Supplement: S1 Fig — (PNG) [file pone.0237515.s001.png]
