# Supplementary material for: Regional variations in Helicobacter pylori infection, gastric atrophy and gastric cancer risk: The ENIGMA study in Chile
Source: PLoS One. 2020 Sep 8;15(9):e0237515. doi: 10.1371/journal.pone.0237515 (PMC7478833; doi:10.1371/journal.pone.0237515)
Supplement: S1 Table — (DOCX) [file pone.0237515.s002.docx]

S1 Table. Demographic characteristics by study site

| Characteristics (%) | | Study site | |  |
| --- | --- | --- | --- | --- |
|  |  | Antofagasta | Valdivia |  |
|  |  | N=690 | N=705 | *P* values† |
|  |  | n(%) | n(%) |  |
| Age |  |  |  |  |
|  | 1-11 years old | 93(14) | 106(15) | 0.71 |
|  | 12-39 years old | 289(42) | 289(41) |  |
|  | 40-70‡ years old | 308(45) | 310(44) |  |
|  |  |  |  |  |
| Gender |  |  |  |  |
|  | Women | 387(56) | 356(51) | 0.04 |
|  | Men | 303(44) | 349(49) |  |
|  |  |  |  |  |
| Ethnicity |  |  |  |  |
|  | Chilean hispanic | 577(84) | 639(91) | <0.001 |
|  | Mapuche | 12(2) | 52(7) |  |
|  | Aymara/Quechua | 22(3) | 0(0) |  |
|  | Other§ | 69(10) | 14(2) |  |
|  | Missing | 10 | 0 |  |
| Body mass index¶ | |  |  |  |
|  | Below 18.5 | 5(1) | 2(0) | 0.59 |
|  | 18.5 to 24.9 | 126(24) | 121(23) |  |
|  | 25.0 to 29.9 | 208(40) | 223(42) |  |
|  | 30 or higher | 180(35) | 190(35) |  |
|  | Missing | 18 | 1 |  |
| Educational level¶ | |  |  |  |
|  | < 8 years | 53(9) | 59(10) |  |
|  | ≥ 8 years | 538(91) | 540(90) | 0.60 |
|  | Missing | 99 | 106 |  |
| † Chi Square or Fisher exact test | |  |  |  |
| ‡ There were one female participant from Antofagasta and one male participant from Valdivia who were 70 years old | | | | |
| § Other includes Colombiano, Diaguita, Peruana and Atacameno | | | | |
| ¶Over 18 year-old | | | | |
