# Supplementary material for: Regional variations in Helicobacter pylori infection, gastric atrophy and gastric cancer risk: The ENIGMA study in Chile
Source: PLoS One. 2020 Sep 8;15(9):e0237515. doi: 10.1371/journal.pone.0237515 (PMC7478833; doi:10.1371/journal.pone.0237515)
Supplement: S2 Table — (DOCX) [file pone.0237515.s003.docx]

**S2 Table**. *H. pylori* seropositivity determined by ELISA and Helicoblot and the frequency of *H. pylori* immunoreactive bands by study site among participants aged 40 and older

|  | | | |
| --- | --- | --- | --- |
|  | Study site | | |
|  | Antofagasta | Valdivia | *P* values^1^ |
|  | N=297  N(%) | N=307  N(%) |  |
|  |  |  |  |
| ***H. pylori* seropositivity** |  |  |  |
| ELISA | 243(82) | 234(76) | 0.06 |
| Helicoblot | 250(84) | 246(80) | 0.20 |
|  |  |  |  |
| **Immunoreactive bands (kDa)** |  |  |  |
| 116 (CagA) | 234(79) | 224(73) | 0.10 |
| 89 (VacA) | 237(80) | 238(78) | 0.50 |
| 37 | 114(39) | 100(33) | 0.14 |
| 35 | 142(48) | 128(42) | 0.13 |
| 30 (UreA) | 209(70) | 213(69) | 0.79 |
| 19.5 | 169(57) | 164(53) | 0.39 |
|  |  |  |  |
| **Immunoreactive bands (kDa) in *H. pylori* positive participants** | | | |
|  | | | |
|  | N=250  N(%) | N=246  N(%) |  |
| 116 (CagA) | 234(94) | 224(91) | 0.29 |
| 89 (VacA) | 237(95) | 237(96) | 0.40 |
| 37 | 114(46) | 100(41) | 0.27 |
| 35 | 142(57) | 127(52) | 0.25 |
| 30 (UreA) | 208(83) | 210(85) | 0.51 |
| 19.5 | 169(68) | 164(67) | 0.83 |
| ^1^ Chi Square |  |  |  |
