# Supplementary material for: Regional variations in Helicobacter pylori infection, gastric atrophy and gastric cancer risk: The ENIGMA study in Chile
Source: PLoS One. 2020 Sep 8;15(9):e0237515. doi: 10.1371/journal.pone.0237515 (PMC7478833; doi:10.1371/journal.pone.0237515)
Supplement: S1 File — (DOCX) [file pone.0237515.s004.docx]

**
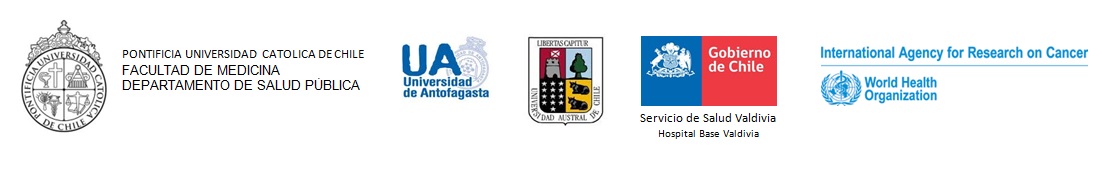
**

Paper identification barcode

# **RISK FACTORS AND COFACTORS INTERVIEW (Nº2A)**

Participants with 12 years of age or older

**PREVALENCE STUDY OF CO-FACTORS ASSOCIATED WITH HELICOBCATER PYLORI INFECTION IN AREAS OF HIGH AND LOW RISK OF GASTRIC CANCER IN CHILE**

**EFR**

| **A** | **DEMOGRAPHIC DATA** | | |
| --- | --- | --- | --- |
|  | Site: (*1. Antofagasta; 2. Valdivia)* | | 🗌 |
|  | Interviewer Code | | 🗌🗌 |
| A1 | Date of Interview *(Day/Month/Year):* | 🗌🗌/🗌🗌/201🗌 | |
| A2 | What is your date of birth? | 🗌🗌/🗌🗌/🗌🗌🗌🗌 | |
| A3 | ¿Were you born in Chile? 1*.Yes (Region/City=_____________________);*  *2. No (Specify country = __________________);*  *8. Don’t know;*  *9. Deny* | | 🗌 |
| A4 | For how long have you lived in this town? *(Years; 88: Don’t know; 99: Refused)* | | 🗌🗌 |
| A5   | Which of the following describes your ethnic origins?  *1. Hispanic Chilean ;*  *2. Mapuche;*  *3. Aymara/Quechua;*  *4. Pascuense (Pascua’s Island);*  *5. European;*  *6. Other (Specify = ______________________)*  *8. Don’t know;*  *9. Refused* | | 🗌 |
| A6   | What is your health system?  *01. Fonasa (public system) 1A. Tipo A; 1B. Tipo B; 1C. Tipo C;*  *1D. Tipo D;*  *02. Isapre (private system);*  *03. FFAA;*  *04. Particular (self-paid);*  *05. Without foresight;*  *06. Other (Specify = ____________________);*  *88. Don’t know;*  *99. Refused* | | 🗌🗌 |
| A7   | What is your educational level?  01. Preschool Education or Preschool Education  02. Preparatory (Old System)  03. Basic Education  04. Humanities (Old System)  05. Scientific-Humanistic Media Education  06. Technical, Commercial, Industrial or Normalistic (Old System)  07. Professional Technical Middle Education  08. Incomplete Technical Training Center (untitled)  09. Complete Technical Training Center (with title)  10. Incomplete Professional Institute (without title)  11. Complete Professional Institute (with title)  12. Incomplete University Education (without title)  13. Complete University Education (with degree)  14. Postgraduate University  88. Don't know;  99. Refused | | 🗌🗌 |
| A8 | How many education years have you completed? | | 🗌🗌 |
| A9 | What is your current work situation?  1. Work / Study;  2. Retired / pensioned;  3. Stay-At-Home (housewife)  4. Unemployed (temporary looking);  5. Permanent unemployed;  8. Don't know;  9. Refused | | 🗌 |
| A10 | What is the area of ​​your work activity?  1. Services;  2. Agricultural;  3. Mining;  4. Construction;  5. Trade;  6. Other (specify = _______________________);  8. Don't know;  9. Refused | | 🗌 |

| A11 | | **Pesticide Exposure History:**  As part of your job, have you ever been exposed to pesticides, either directly (as an applicator) or indirectly?  1. Yes;  2. No;  8. Don't know;  9. Refused | | | | | | | 🗌 |
| --- | --- | --- | --- | --- | --- | --- | --- | --- | --- |
|  | What was / is the pesticide?  Specify | | How were you exposed?  *1. Indirect;*  *2. Direct;*  *8. Don’t know;*  *9. Refused* | When did the pesticide exposure began (age)?  *88. Don’t know;*  *99. Refused* | When did the pesticide exposure stop (age)?  *88. Don’t know;*  *99. Refused* | In a year, you were exposed:  *1. Constantly*  *2.Intermittently*  *8. Don’t know;*  *9. Refused* | On average in a year, how many months were you exposed to pesticides?  *88. Don’t know;*  *99. Refused* | How were the pesticides applied?  *1. Plane;*  *2. Tractor;*  *3. Hand sprayer;*  *4. Back sprayer;*  *8. Don’t know;*  *9. Refused* | In what type of crops?  Specify |
| A11a |  | | 🗌 | 🗌🗌 | 🗌🗌 | 🗌 | 🗌🗌 | 🗌 |  |
| A11b |  | | 🗌 | 🗌🗌 | 🗌🗌 | 🗌 | 🗌🗌 | 🗌 |  |
| A11c |  | | 🗌 | 🗌🗌 | 🗌🗌 | 🗌 | 🗌🗌 | 🗌 |  |

| **B** | **TOBACCO USE HISTORY** | | | |
| --- | --- | --- | --- | --- |
| B1 | Do you smoke or have you smoked a total of 100 cigarettes/5 packs or more in your life ?  *1. Yes;*  *2. No* ***(GO TO SECTION C)****;*  *8. Don’t know;*  *9. Refused)* | | | 🗌 |
| B2 | How old were you when you started smoking at least one cigarette daily?  *88/8888 Don’t know;*  *99/9999 Refused* | AGE YOU STARTED: 🗌🗌  OR YEAR: 🗌🗌🗌🗌 | | |
| B3 | Are you currently smoking at least one cigarette daily?  *1. Yes* ***(GO TO B5)****;*  *2. No;*  *8. Don’t know;*  *9. Refused* | | | 🗌 |
| B4 | How old were you when you stopped smoking regularly?  *88/8888. Don’t know;*  *99/9999. Refused* | AGE YOU STOPPED: 🗌🗌  OR YEAR: 🗌🗌🗌🗌 | | |
| B5 | Think about all the years you smoked, how many cigarettes did you smoke on a day ?  *888. Don’t know;*  *999. Refused* | | 🗌🗌🗌 | |

| **C** | **MEDICAL HISTORY**  **If any question from C9 to C12 is Yes recommend medical evaluation as soon as possible** | | | |
| --- | --- | --- | --- | --- |
|  | **Symptom**  In the last 3 months, how often did you have the following symptoms? | **Frequency**  *0. Did not have;*  *1. Less than one day a month;*  *2. One day a month;*  *3. 2-3 days a month;*  *4. One day a week;*  *5. More than one day a week*  *8. Don’t know*  *9. Refused* | **Intensity**   1. *Didn’t have;* 2. *Very slight;* 3. *Mild;* 4. *Moderate;* 5. *Intense;* 6. *Very intense*   *8. Don’t know*  *9. Refused* | |
| C2a | Burning sensation behind the breastbone: | 🗌 | 🗌 | |
| C2b | Pain behind the breastbone: | 🗌 | 🗌 | |
| C2c | Burning sensation in the center of the abdomen above the navel: | 🗌 | 🗌 | |
| C2d | Pain in the center of the abdomen above the navel: | 🗌 | 🗌 | |
| C2e | Acid taste in the mouth: | 🗌 | 🗌 | |
| C2f | Movement of food that is returned to the mouth: | 🗌 | 🗌 | |
| C2g | Nausea: | 🗌 | 🗌 | |
| C2h | Annoying belching | 🗌 | 🗌 | |
| C2i | Did you feel too satisfied after eating a normal amount of food? | 🗌 | 🗌 | |
| C4 | Have you had any of the symptoms described above for 15 days or more?  *1. Yes;*  *2. No;*  *8. Don’t know;*  *9. Refused* | | | 🗌 |
| C5 | In the last 3 months, how often did you have diarrhea (3 or more liquid stools in 24 hours or 1 bloody stool in 24 hours)?  *1. Nunca o rara vez* ***(GO TO C7)****;*  *2. Sometimes;*  *3. Often;*  *4. Most of the time;*  *5. Always;*  *8. Don’t know;*  *9. Refused* | | | 🗌 |
| C6 | When was the last time you had diarrhea (last three days)?  *1. Today;*  *2. Yesterday;*  *3. The day before yesterday;*  *8. Don’t know;*  *9. Refused* | | | 🗌 |
| C7 | In the last 3 months, how often did you vomit?  *1. Never or rarely;*  *2. Sometimes;*  *3. Often;*  *4. Most of the time;*  *5. Always;*  *8. Don’t know;*  *9. Refused* | | | 🗌 |
| C8 | In the last 3 months, have you felt a decrease in your appetite?  *1. Yes;*  *2. No;*  *8. Don’t know;*  *9. Refused* | | |  |
| C9 | Have you ever had vomiting or bloody stools?  *1. Yes;*  *2. No;*  *8. Don’t know;*  *9. Refused* | | | 🗌 |
| C10 | In the last 3 months, have you noticed and unintentional weight loss?  *1. Yes;*  *2. No;*  *8. Don’t know;*  *9. Refused* | | | 🗌 |
| C11 | In the last 3 months, have you had difficulty swallowing food, feeling that it does not pass ?  *1. Yes;*  *2. No;*  *8. Don’t know;*  *9. Refused* | | | 🗌 |
| C12 | In the last 3 months, have you noticed the presence of any mass or lump in your abdomen?  *1. Yes;*  *2. No;*  *8. Don’t know;*  *9. Refused* | | | 🗌 |

| C11 | **Medical History:** | | | |
| --- | --- | --- | --- | --- |
|  | *Ask about each condition (from a through k), is the answer is “yes”* ***Go to the next column*** *for each condition. If the answer is “No”* ***go to the next condition.*** | Have you been diagnosed with any of the following conditions by your treating physician?  *1. Yes;*  *2. No;*  *8. Don’t know;*  *9. Refused* | How old were you when the condition was diagnosed?  Age  *88. Don’t know;*  *99. Refused* | Have you ever been hospitalized for this condition?  *1. Yes = amount of times ________;*  *2. No;*  *8. Don’t know;*  *9. Refused* |
| C11a | Asthma | 🗌 | 🗌🗌 | 🗌 ___ |
| C11b | Tuberculosis | 🗌 | 🗌🗌 | 🗌 ___ |
| C11c | Anemia | 🗌 | 🗌🗌 | 🗌 ___ |
| C11d | Inflammatory colon disease, or ulcerative colitis or Crohn's disease | 🗌 | 🗌🗌 | 🗌 ___ |
| C11e | Intestinal paracites | 🗌 | 🗌🗌 | 🗌 ___ |
| C11f | Diabetes Mellitus | 🗌 | 🗌🗌 | 🗌 ___ |
| C11g | Peptic ulcer | 🗌 | 🗌🗌 | 🗌 ___ |
| C11h | Helicobacter pylori infection | 🗌 | 🗌🗌 | 🗌 ___ |
| C11i | Gallstones | 🗌 | 🗌🗌 | 🗌 ___ |
| C11j | Acute cholecystitis | 🗌 | 🗌🗌 | 🗌 ___ |
| C11k | Rheumatoid arthritis | 🗌 | 🗌🗌 | 🗌 ___ |

| **D** | **HISTORY OF DISEASES IN THE FAMILY** | | | |
| --- | --- | --- | --- | --- |
| D1 | Has an immediate family member been diagnosed with stomach cancer?  *1. Yes;*  *2. No (GO TO E);*  *8. Don’t know;*  *9. Refused* | | | 🗌 |
| D2 | Which of your relatives had stomach cancer?  *1. Yes;*  *2. No;*  *8. Don’t know;*  *9. Refused* | | At what age did they have stomach cancer?  Age  *88. Don’t know;*  *99. Refused* | |
| D2a | Biological mother | 🗌 | 🗌🗌 | |
| D2b | Biological father | 🗌 | 🗌🗌 | |
| D2c | Brothers/sisters | 🗌 | 🗌🗌 | |
| D2d | Biological children | 🗌 | 🗌🗌 | |
| D2e | Grandparents | 🗌 | 🗌🗌 | |

| **E** | **USE OF MEDICINE AND ANTIBIOTICS** | | | | | | | | |
| --- | --- | --- | --- | --- | --- | --- | --- | --- | --- |
| The following questions are about the use of medications prescribed by a doctor or not prescribed.  ASK ABOUT THE CONSUMPTION OF EACH MEDICATION (E1a.and E1b), IF THE ANSWER IS “YES”, *GO TO THE NEXT COLUMNS* FOR EACH MEDICATION AND THEN *GO TO THE NEXT MEDICATION*. IF THE ANSWER IS “NO”, “DON’T KNOW” OR “REFUSED” *GO TO THE NEXT MEDICATION*. | | | | | | | | | |
|  | | Have you consumed regularly, that is 2 or more times a week for 2 months or more, any of the following medications?  *1. Yes;*  *2. No;*  *8. Don’t know;*  *9. Refused* | | | For how long did you regularly consume? | | Are you currently taking this medicine?  *1. Yes;*  *2. No;*  *8. Don’t know;*  *9. Refused* | | |
|  |  |  |  |  | *(Number)*  *88. Don’t know*  *99. Refused* | *1. Days;*  *2. Weeks;*  *3. Months*  *4. Year;*  *8. Don’t know*  *9. Refused* |  |  |  |
| E1a | | Aspirin | | 🗌 | 🗌🗌 | 🗌 | 🗌 | | |
| E1b | | Nonsteroidal anti-inflammatory drugs (NSAIDs), pain relief medications (exclude acetaminophen) | | 🗌 | 🗌🗌 | 🗌 | 🗌 | | |
| E2 | | | Was any of the medications or injections you received in the past year an antibiotic or an antiparasitic?  *1. Yes;*  *2. No (GO TO F);*  *8. Don’t know;*  *9. Refused* | | | | | 🗌 | |
| E3 | | | How many times have you received antibiotics or dewormers in the last year?  *1. One time;*  *2. Two times;*  *3. Three or more times;*  *8. Don’t know;*  *9. Refused* | | | | | 🗌 | |
| E4 | | | When was the last time you received antibiotics or dewormers?  *1. Less than a month ago;*  *2. 1 or 2 months ago;*  *3. 3 or 4 months ago;*  *4. 4 or 5 months ago;*  *5. 6 months ago or more;*  *8. Don’t know;*  *9. Refused* | | | | | 🗌 | |
| E5 | | | For what reason did you last receive antibiotics or dewormers?  1. Flu, bronchitis, sore throat, other respiratory infection;  2. Diarrhea, gastritis, another digestive problem;  3. Skin infection;  4. Other reason, specify *= _______________;*  *8. Don’t know;*  *9. Refused* | | | | | 🗌 | |
| E6 | | | If you know the name of the antibiotics or dewormers you took, please indicate them: | | | | | | |
| E7 | | | Have you ever been treated for Helicobacter pylori in your life?  *1. Yes (How long ago* 🗌🗌 *years and* 🗌🗌 *months);*  *2. No;*  *8. Don’t know;*  *9. Refused* | | | | | | 🗌 |

| **F** | **DIETARY HISTORY** | | | | |
| --- | --- | --- | --- | --- | --- |
| These questions are about the different types of food that you ate or drank in the past 3 years and 20 years ago. In your answer, please include food at home, work, school, restaurants, and anywhere else. | | | | | |
|  |  | **Actually** | | **20 years ago** | |
|  | In the past three years, how many times a day, week, or month did you eat or drink:  (write down the foods that the subject names) | **Number of times** | **Frequency**  *0. Never;*  *1. Day;*  *2. Week;*  *3. Month;*  *8. Don’t know;*  *9. Refused* | **Number of times** | **Frequency**  *0. Never;*  *1. Day;*  *2. Week;*  *3. Month;*  *8. Don’t know;*  *9. Refused* |
| F1 | Fresh green chilli peppers? | 🗌🗌 | 🗌 | 🗌🗌 | 🗌 |
| F2 | Fresh red chilli peppers? | 🗌🗌 | 🗌 | 🗌🗌 | 🗌 |
| F3 | Red chili pepper powder? | 🗌🗌 | 🗌 | 🗌🗌 | 🗌 |
| F4 | Red chili pepper paste? | 🗌🗌 | 🗌 | 🗌🗌 | 🗌 |
| F5 | Chilli merken’ | 🗌🗌 | 🗌 | 🗌🗌 | 🗌 |
| F6 | Fresh fruits (apples, oranges, etc)? | 🗌🗌 | 🗌 | 🗌🗌 | 🗌 |
| F7 | Green vegetables (Broccoli, cabbage, etc)? | 🗌🗌 | 🗌 | 🗌🗌 | 🗌 |
| F8 | Other vegetables (carrots, beans, etc)? | 🗌🗌 | 🗌 | 🗌🗌 | 🗌 |
| F9 | Fatty fish (salmon, grouper)? | 🗌🗌 | 🗌 | 🗌🗌 | 🗌 |
| F10 | Other fish (hake, pippin, etc.)? | 🗌🗌 | 🗌 | 🗌🗌 | 🗌 |
| F11 | Canned fish or seafood? | 🗌🗌 | 🗌 | 🗌🗌 | 🗌 |
| F12 | Meat (Pork or Cow)? | 🗌🗌 | 🗌 | 🗌🗌 | 🗌 |
| F13 | Chicken or Turkey | 🗌🗌 | 🗌 | 🗌🗌 | 🗌 |
| F14 | Products derived from meat such as sausages, ham, etc? | 🗌🗌 | 🗌 | 🗌🗌 | 🗌 |
| F15 | Eggs? | 🗌🗌 | 🗌 | 🗌🗌 | 🗌 |
| F16 | Cheese? | 🗌🗌 | 🗌 | 🗌🗌 | 🗌 |
| F17 | Wholemeal bread? | 🗌🗌 | 🗌 | 🗌🗌 | 🗌 |
| F18 | Other wholemeal foods (rice, noodles, etc)? | 🗌🗌 | 🗌 | 🗌🗌 | 🗌 |
| F19 | High fiber cereals? | 🗌🗌 | 🗌 | 🗌🗌 | 🗌 |
| F20 | Salty or smoked foods? | 🗌🗌 | 🗌 | 🗌🗌 | 🗌 |
| F21 | Fried foods? | 🗌🗌 | 🗌 | 🗌🗌 | 🗌 |
| F23 | Potatoes with peel? | 🗌🗌 | 🗌 | 🗌🗌 | 🗌 |
| F24 | Potatoes without peel? | 🗌🗌 | 🗌 | 🗌🗌 | 🗌 |
| F22 | How often do you add salt to food before trying?  *0. Never or rarely;*  *1. Sometimes;*  *2. Often;*  *3. Most of the time;*  *4. Always;*  *8. Don’t know;*  *9. Refused* | | | | 🗌 |

| **G** | **ALCOHOL CONSUMPTION** | | | | | |
| --- | --- | --- | --- | --- | --- | --- |
| G1 | During your life, have you ever consumed alcoholic beverages?  *1. Yes;*  *2. No (GO TO H);*  *8. Don’t know;*  *9. Refused* | | | | | 🗌 |
| G2 |  | **In the last 3 years** | | **20 years ago** | | |
|  | **Beverage** | Number of times  *Never: 00*  *Don’t know: 88*  *Refused: 99* | Frequency  *0. Never;*  *1. Day;*  *2. Week;*  *3. Month;*  *8. Don’t know;*  *9. Refused* | Number of times  *Never: 00*  *Don’t know: 88*  *Refused: 99* | Frequency  *0. Never;*  *1. Day;*  *2. Week;*  *3. Month;*  *8. Don’t know;*  *9. Refused* | |
| G2a | ¿Beer (350 ml serving, i.e. a regular can or bottle )? | 🗌🗌 | 🗌 | 🗌🗌 | 🗌 | |
| G2b | ¿Wine (150 ml serving)? | 🗌🗌 | 🗌 | 🗌🗌 | 🗌 | |
| G2c | ¿Other liquors (pisco, tequila, vodka) a measure is 30 ml? | 🗌🗌 | 🗌 | 🗌🗌 | 🗌 | |
| G3 | Would you say that you don't drink regularly but consume large amounts of alcohol from time to time in a short period of time?  *1. Yes;*  *2. No;*  *8. Don’t know****;***  *9. Refused* | | | | | 🗌 |
| G4   | Have you ever felt that when you drink alcohol your face temperature increases or have you been told that when you drink alcohol you blush or turn red?  *1. Yes;*  *2. No;*  *8. Don’t know****;***  *9. Refused* | | | | | 🗌 |

| **H** | **EVALUATION OF THE QUALITY OF THE INTERVIEW** | |  |
| --- | --- | --- | --- |
| **ESTA SECCION DEBE SER RESPONDIDA POR EL ENTREVISTADOR** | | | THIS SECTION MUST BE ANSWERED BY THE INTERVIEWER |
| H1 | The participant's cooperation was:  1. Poor;  2. Regular;  3. Good;  4. very good | 🗌 |  |
| H2 | The overall quality of the interview was:  1. Unreliable;  2. Reliable in general;  3. Very reliable | 🗌 |  |
| H2a | If the quality of the interview was UNRELIABLE, indicate the reason:  The participant:  1. Did not understand the terms used well;  2. Didn't want to be more specific;  3. Was bored, without interest;  4. Was angry or depressed;  5. Did not hear or speak clearly;  6. There were many interruptions;  7. There was not enough privacy;  8. Was ashamed;  9. Was sick | 🗌 |  |
| H3 | Any other comments about the interview: | |  |

| **I** | **ANTHROPOMETRIC MEASURES** | |
| --- | --- | --- |
| PROCEED TO TAKE THE PARTICIPANT'S SIZE, WEIGHT AND ABDOMINAL CIRCUMFERENCE, IF HE/SHE IS OVER 18 YEARS OF AGE, ALSO TAKE THE BLOOD PRESSURE AND GIVE RECOMMENDATIONS IF NECESSARY. | | |
| I1 | Size: *(cm)* | 🗌🗌🗌 |
| I2 | Weight*: (Kg)* | 🗌🗌🗌.🗌 |
| I3 | Abdominal circumference: *(cm)* | 🗌🗌🗌 |
| I4 | Blood pressure: (mmHg) Systolic / Diastolic  *888/888 if underage* | 🗌🗌🗌/🗌🗌🗌 |
| I5 | Requires recommendations:  *1. Yes;*  *2. No* | 🗌 |

| **J** | **DATA FOR FUTURE CONTACT IF NECESSARY** | | |
| --- | --- | --- | --- |
| J1. | **Personal contact information** | | |
| J1.a. Home telephone number:  \|__\|__\|__\|__\|__\|__\|__\|__\|__\| | | J1.b. Cellphone number:  \|__\|__\|__\|__\|__\|__\|__\|__\|__\| | |
| J1.c. Address: **_____________________________________________________________________________________________________________**  **________________________________________________________________________________________________________________________________** | | | |
| J2. | **Alternative contact information** | | |
| J2.a. Name of a contact person who does not live in the same house  **_______________________________________________________________________________________________________________________________** | | | |
| J2.b. Relationship with the participant?  1. Mother / Father  2. Child  3. Uncle / Aunt  4. Grandfather  5. Friend  6 Other If other, specify________________________________________ | | | 🗌 |
| J2.c. Home telephone numer:  \|__\|__\|__\|__\|__\|__\|__\|__\|__\| | | J2.d. Cellphone number:  \|__\|__\|__\|__\|__\|__\|__\|__\|__\| | |
| J2.e. Address: **_____________________________________________________________________________________________________________**  **________________________________________________________________________________________________________________________________** | | | |
